# The targeted histone deacetylase inhibitor tefinostat (CHR-2845) shows selective *in vitro* efficacy in monocytoid-lineage leukaemias

**DOI:** 10.18632/oncotarget.7692

**Published:** 2016-02-25

**Authors:** Joanna Zabkiewicz, Marie Gilmour, Robert Hills, Pares Vyas, Elizabeth Bone, Alan Davidson, Alan Burnett, Steven Knapper

**Affiliations:** ^1^ Department of Haematology, Experimental Cancer Medicine Centre (ECMC), Institute of Cancer and Genetics, School of Medicine, Cardiff University, Cardiff, UK; ^2^ Weatherall Institute of Molecular Medicine, University of Oxford, Oxford, UK; ^3^ Chroma Therapeutics, Abingdon, UK

**Keywords:** AML, HDACi, CMML, tefinostat, hCE-1

## Abstract

Tefinostat (CHR-2845) is a novel monocyte/macrophage-targeted histone deacetylase (HDAC) inhibitor which is cleaved into its active acid by the intracellular esterase human carboxylesterase-1 (hCE-1). The *in vitro* efficacy of tefinostat was characterised in cell lines and in a cohort of 73 primary AML and CMML samples. Dose-dependent induction of apoptosis and significant growth inhibitory effects were seen in myelomonocytic (M4), monocytic/monoblastic (M5) and CMML samples in comparison to non-monocytoid AML sub-types (*p* = 0.007). Importantly, no growth inhibitory effects were seen in normal bone marrow CD34^+^ cells exposed to AML-toxic doses of tefinostat in clonogenic assays. Expression of hCE-1 was measured by intracellular flow cytometry and immunoblotting across the cohort, with highest levels seen in M5 AML patients. hCE-1 levels correlated with significantly increased tefinostat sensitivity (low EC50) as measured by growth inhibition assays (*p* = 0.001) and concomitant elevation of the mature monocytoid marker CD14^+^. Strong induction of intracellular histone protein acetylation was observed in tefinostat-responsive samples, as were high levels of the DNA damage sensor γ-H2A.X, highlighting potential biomarkers of patient responsiveness. Synergistic interaction between tefinostat and the current standard treatment cytarabine was demonstrated in dose response and clonogenic assays using simultaneous drug addition in primary samples (median Combination Index value = 0.51). These data provide a strong rationale for the further clinical evaluation of tefinostat in monocytoid-lineage haematological neoplasms including CMML and monocyte-lineage AMLs.

## INTRODUCTION

Monocytoid-lineage leukaemias encompass the monocytoid subtypes of acute myeloid leukaemia (AML) (acute myelomonocytic leukaemia [previously FAB type M4] and acute monoblastic/monocytic leukaemia [M5]), as well as chronic myelomonocytic leukaemia (CMML) and juvenile myelomonocytic leukaemia (JMML) [1, 2]. Although the overall prognosis of AML has improved gradually over the last 40 years, the majority of patients continue to succumb to the disease with clinical prospects remaining particularly bleak for older patients; there has been little change to standard chemotherapeutic treatment approaches over this time [3]. CMML is a neoplasm that is classified within the WHO sub-categorisation of ‘myelodysplastic / myeloproliferative neoplasms’ [2] that has a high median age of presentation (70–75 years), a median survival of only 11–17 months and currently very limited treatment options [4, 5]. There is a pressing need to exploit advances made in the understanding of the pathogenetic mechanisms that underpin monocytoid malignancies by developing novel therapeutic agents, applicable ideally to the treatment of patients of all ages, that are able to effectively deliver targeted effects to malignant cell populations while avoiding significant systemic toxicity.

Histone acetylation is an important molecular modification used to regulate gene transcription that affects many cellular processes including cell proliferation, differentiation, DNA repair, cell survival and angiogenesis [6–8]. Histone deacetylases (HDACs) are a family of epigenetic modifiers that alter chromatin structure by removing lysine acetylation from histones, resulting in a transcriptionally-closed state and subsequent gene silencing or loss of expression. Alterations in epigenetic programming have been commonly reported in the initiation, progression and maintenance of cancer; and aberrant localization of HDACs and resultant promoter silencing has been implicated in several malignancies [7], [9] including AML and the myelodysplastic syndromes (MDS) [10–12] and has been reported in connection with common oncogenic fusion proteins such as AML1ETO (t8:21), and Inv16 (core binding protein) abnormalities [13]. Myelomonocytoid and monocytoid / monoblastic AML (FAB M4 and M5) accounts for approximately 25% of total AML cases [44]; these cases have a distinct clinical profile with frequent extramedullary manifestations and leucocytosis along with emerging associations with abnormalities in epigenetic regulation in the form of *DNMT3A* mutations [45, 46].

Given the reversible nature of acetylation modifications, therapeutic targeting of HDACs has been an active area of drug development with the promise of correcting the effects of aberrant gene expression [14]. HDAC inhibitors may exert their activity by multiple mechanisms of action including: cell differentiation, DNA repair inhibition [15], induction of reactive oxygen species [16], and replication stalling [17]. Clinical trials of several HDAC inhibitors including valproic acid, vorinostat, romidepsin, belinostat and panabinostat have been conducted in both solid tumours and haematological malignancies including AML, MDS and CMML patients [18–22]. In general, reported clinical responses to single-agent HDAC inhibitory therapy have been modest with dose escalation of HDAC inhibitors being limited by a relatively restricted therapeutic window. Off-target effects of HDAC inhibition have been associated with significant systemic toxicities including gastrointestinal disturbances, thrombocytopenia, fatigue and insomnia which have limited the wider clinical uptake of these agents. It is highly desirable to develop mechanisms through which HDAC inhibitory activity can be more-selectively concentrated within tumour cells while sparing non-disease cell populations.

Tefinostat (CHR-2845) is a novel pan HDAC inhibitor which is cleaved to an active acid, CHR-2847, by the intracellular esterase human carboxylesterase-1 (hCE-1), the expression of which is limited to cells of monocytoid lineage and some hepatocytes, allowing selective accumulation of active drug within monocytoid cells. [23]. A phase I dose escalation study of tefinostat in patients with relapsed/refractory haematological malignancies demonstrated early signs of clinical efficacy without any dose limiting toxicity. [23].

We examined the pre-clinical activity of tefinostat in a large cohort of primary AML and CMML patient samples in order to assess lineage specific activity, potential therapeutic window and combination studies with Cytarabine to build a rationale for future therapeutic evaluation in monocytoid leukaemias.

## RESULTS

### Monocytoid leukaemias show selective high sensitivity to tefinostat

**Figure 1:**
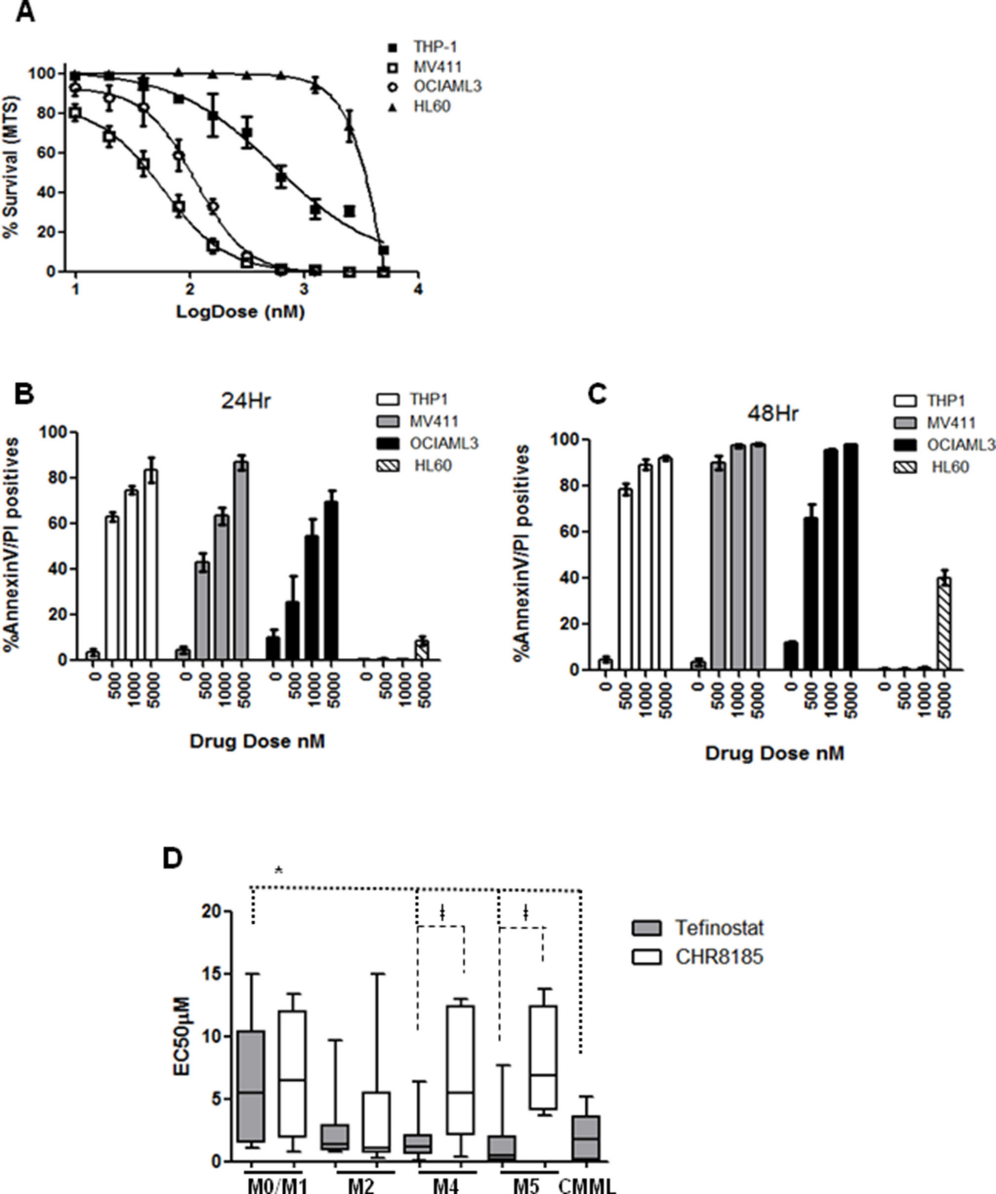
Monocytoid leukaemias show selective high sensitivity to Tefinostat (**A**) Dose response plot for AML cell lines HL60 (M2), MV411 (M4), OCIAML3 (M4) and THP-1 (M5) treated with serial dilutions of Tefinostat. Tefinostat-induced Annexin V/PI incorporation measured by flow cytometry in THP1, MV411, OCIAML3 and HL60 at (**B**) 24 Hrs and (**C**) 48 Hrs post treatment. (**D**) Box and whiskers plots showing range of FAB type-specific MTS-derived EC50s in response to Tefinostat and the comparative non hCE-1-dependent t-butyl analogue CHR8185 in a cohort of 66 primary AML and 7 primary CMML samples. (Tefinostat EC50 M4/M5/CMML vs. M0/M1 *p = 0.009 spearman's correlation. Tefinostat vs. t-butyl analogue CHR8185, only significant in M4/M5 groups †*p* < 0.007).

The *in vitro* efficacy of tefinostat was first assessed by MTS cell viability assay in AML cell lines HL60 (M2 FAB type), MV411 (M4, FLT3-ITD), OCIAML3 (M4 NPM1mut) and THP1 (M5) (EC_50_ = 2300 nM +/−226 vs. 57 nM +/−6.2 vs. 110 nM +/− vs. 560 nM +/−17.12 respectively, Figure 1A). Annexin V/PI incorporation showed strong apoptotic induction in myelo-monocytic cell lines THP1, MV411 (FLT3-ITD) and OCIAML3 within 24 hours of tefinostat treatment that was only reached in non-monocytic HL60 cells at much higher drug concentrations (Figure 1B–1C). Dose response to tefinostat was assessed in a cohort of 66 primary AML and 7 primary CMML samples (Ave EC_50_ 2.7 μM +/− 3.1). Significant growth inhibitory effects were seen in M4 (myelomonocytic)/M5 (monocytic / monoblastic) AMLs and CMML samples with lower EC50s in comparison to non-M4/M5 AML FAB types (mean EC_50_ M4/M5 = 1.1 μM +/−1.8, CMML = 1.9 +/−1.6 vs. M0/M1 = 5.1 μM +/−4.7 respectively, *p = 0.009 spearman's correlation, Figure 1D). This selectivity between M0/M1 and M4/M5 FAB groups was abolished when the t-butyl tefinostat analogue CHR-8185 (which is not cleaved by hCE-1) was substituted as an alternative HDACi, further supporting the monocytoid selectivity of tefinostat. M2 FAB type AMLs displayed a wide range of sensitivity of response to tefinostat; overall responses of M2 samples were not significantly different from the M4/M5 sub-groups. Importantly, there was no differential response between tefinostat and CHR8185 in the M2 subgroup, suggesting responses to be non hCE-1 mediated in this group (Figure 1D).

Further analysis of the relationship between *in vitro* tefinostat sensitivity (log_10_(EC_50_) and patient characteristics revealed no significant differences in drug efficacy according to other disease parameters including clinical outcome, presenting cytogenetics and FLT3/NPM1 mutational status.(Supplementary Table S1).

### Monocytic targeting of HDACi therapy spares normal bone marrow progenitor cells

**Figure 2:**
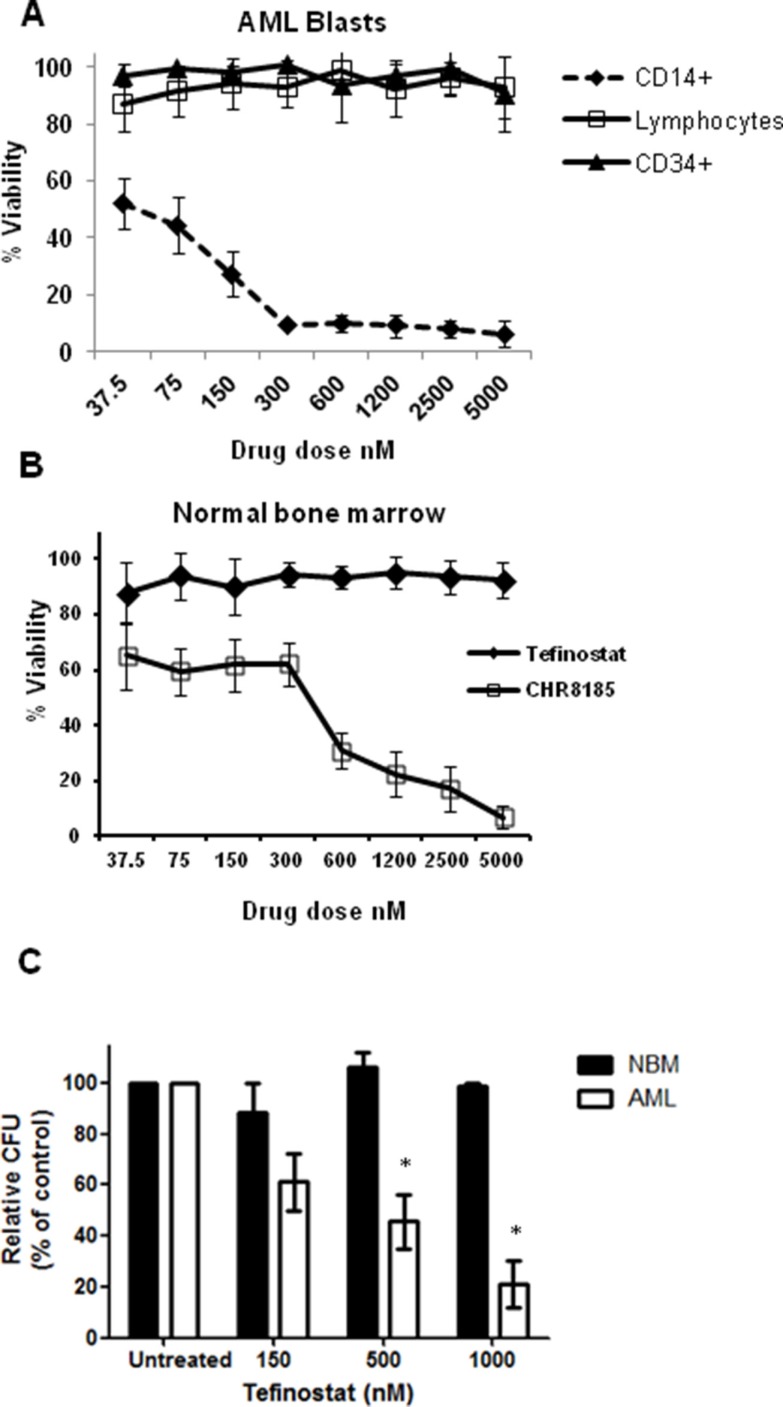
Monocytic targeting of HDACi therapy spares normal bone marrow progenitor cells (**A**) Flow cytometric analysis of viability of cell subpopulations in primary AML samples in response to Tefinostat dosing measured by 7AAD exclusion (*n* = 8). (**B**) Dose response effects of Tefinostat and CHR8185 in NBM cells (*n* = 4) measured by 7AAD exclusion. (**C**) Tefinostat effects on Colony forming units (CFUs) in NBM CD34^+^ cells (no significant difference, *n* = 4) and primary AML blasts (*p < 0.02 MWU, *n* = 6) following 14 days culture on methocult.

Analysis of selective response to tefinostat in sub-populations of primary cells was carried out using flow cytometric 7AAD exclusion assays. CD14^+^ AML blasts were highly sensitive to tefinostat treatment in contrast to CD34^+^/CD14^−^ AML blasts and CD45^high^ lymphocyte populations (Figure 2A). This sensitivity was significantly reduced in when equivalent concentrations of the non-hCE-1-dependent analogue CHR8185 were used, demonstrating the myelo-monocytic specificity of Tefinostat (Supplementary Figure S1). Importantly, no growth inhibitory effects were seen in normal bone marrow (NBM) CD34^+^ cells exposed to AML-toxic doses of tefinostat while, in comparison, equivalent concentrations of CHR-8185 caused considerable cytotoxicity (Figure 2B). Exposure of NBM progenitor cells to increasing concentrations of tefinostat in longer-term colony assays failed to reduce colony forming units in comparison to vehicle-treated controls and AML treated samples (pre-selected for high CD14+ percentage), which showed significant reduction in colony formation (Figure 2C, *p* < 0.02).

### hCE-1 expression levels dictate efficacy of tefinostat in monocytic leukaemias

**Figure 3:**
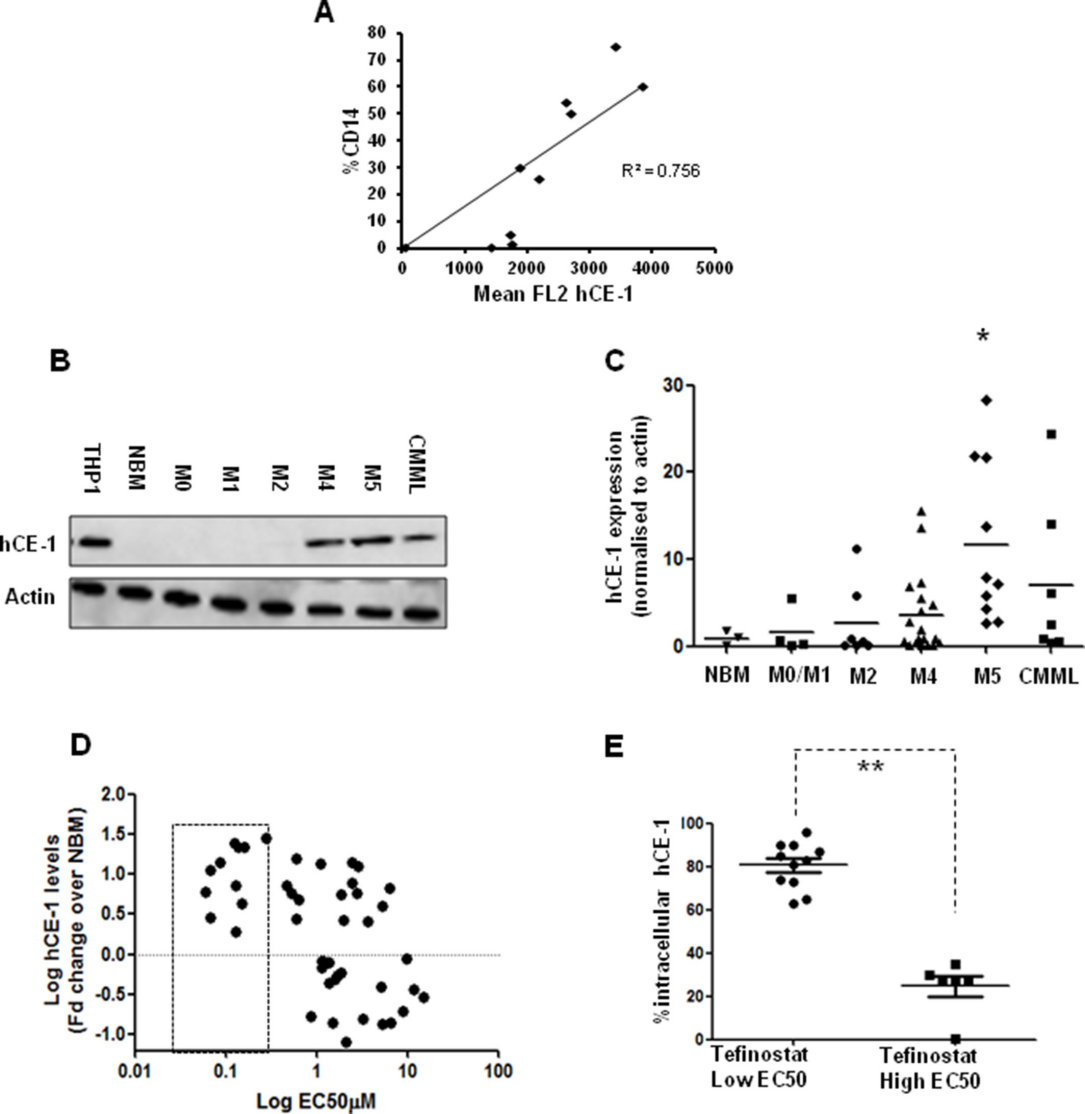
hCE-1 expression levels dictate efficacy of Tefinostat in monocytic leukaemias (**A**) Correlation plot of CD14^+^ expression vs. hCE-1 mean fluorescence measured by intracellular flow in primary AML samples (*n* = 10) (**B**) Representative western blot of hCE-1 expression in NBM, AML and CMML patient samples. (**C**) Comparison of hCE-1 protein levels across FAB types (NBM vs. M5 *p = 0.01 Wilcoxon rank sum). (**D**) Correlation plot of hCE-1 protein levels measured by western blot (fold change relative to NBM levels, *n* = 3) compared to tefinostat sensitivity (EC_50_) in a cohort of 40 primary AML and 7 CMML samples, *p* = 0.001 Spearman's correlation per 10 fold increase in EC50). Dotted box represents low EC50 samples. (**E**) Monocytoid intracellular hCE-1 levels in tefinostat-sensitive and tefinostat-resistant primary samples gated by CD14+/CD64+ (EC_50_ high (*n* = 6) and low patients (*n* = 11), **p < 0.001 MWU).

hCE-1 expression in primary samples was quantified by intracellular flow cytometry in combination with cell surface markers CD14, CD64 and CD45 to allow identification of hCE-1 levels in different cellular sub-populations (Supplementary Figure S2). Significant correlation was seen between levels of the mature monocytic marker CD14^+^ and hCE-1 expression in AML samples (Figure 3A). Further analysis of a larger cohort of 40 primary AMLs and 7 CMMLs by western blot confirmed highest hCE-1 levels in myelomonocytic and monocytic FAB types, with M5 AMLs displaying significant overexpression in comparison to NBM levels (Figure 3B–3C, *p* = 0.01). This observation was validated by microarray analysis of hCE-1 mRNA in a further 130 AML samples, with M4/M5 AMLs showing significant overexpression compared to NBM CD34^+^ cells (Supplementary Figure S3). High hCE-1 levels was associated with low EC50 values to tefinostat across the cohort as measured by western blotting (Figure 3D *p* < 0.001). To supplement western blot expression levels, intracellular flow analysis of hCE-1 in a smaller cohort of M2, M4 and M5 monocytoid AML samples was divided into tefinostat-sensitive primary AMLs (EC_50_ < 1 μM Ave) and tefinostat-resistant samples (EC_50_ > 2.5 μM). Significantly higher intracellular flow levels of hCE-1 were observed in tefinostat-sensitive AMLs compared to resistant samples (Figure 3E *p* < 0.001).

### Increases in intracellular acetylation and DNA damage induction are biomarkers of tefinostat efficacy

**Figure 4:**
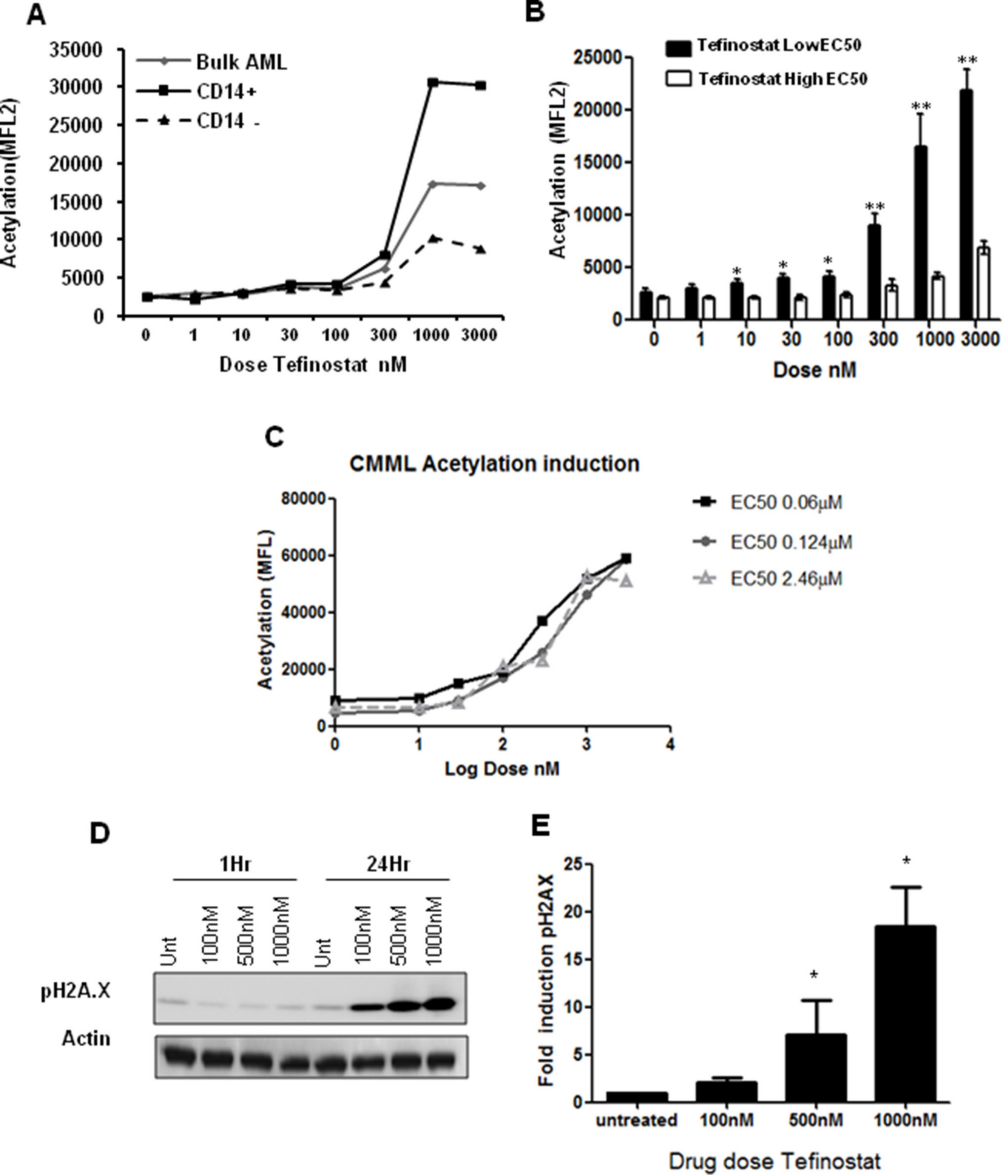
Increases in intracellular acetylation and DNA damage induction are biomarkers of Tefinostat efficacy (**A**) Tefinostat dose-dependent intracellular acetylation staining in a representative primary AML sample using acetylated lysine monoclonal antibody and sub-population analysis by flow cytometry. (**B**) Acetylation induction in Tefinostat sensitive (low EC_50_/CD14^+^ black bars, *n* = 8) compared to insensitive (high EC_50_, white bars, *n* = 5) primary AML samples, *p < 0.05, **p < 0.005. (**C**) Tefinostat dose-dependent intracellular acetylation induction in primary CMML samples (*n* = 3). All samples exhibit > 80% CD14+ and > 70% hCE-1 expression. (**D**) Representative western blot of phospho-H2A.X induction by tefinostat at 1 and 24 hours post-treatment. (**E**) Western blot quantification of dose-dependent tefinostat-induced γ-H2A.X induction at 24 hrs compared to vehicle treated control (*n* = 9 AML samples, *p < 0.01 Kruskal Wallis).

In order to identify biomarkers of tefinostat sensitivity in AML blasts we undertook sub-population analysis by flow cytometry to look for differential effects on intracellular acetylation. CD14-expressing cells showed a maximum induction of intracellular protein acetylation at nanomolar tefinostat concentrations after 6 hours of drug exposure (Figure 4A). This analysis was extended to compare tefinostat sensitive (*n* = 8, EC50 < 1 μM) and resistant AML samples (*n* = 5, EC50 > 2.5 μM); significant acetylation was induced at low nanomolar doses in low EC_50_ samples, but was absent in those patients with resistance to tefinostat (Figure 4B). Strong acetylation induction was also observed in several CMML patient samples (Figure 4C), although this appeared to be less variable with Tefinostat sensitivity. Tefinostat-sensitive samples also showed strong phosphorylation induction of the cell cycle arrest and DNA double strand break damage sensor protein γ-H2A.X, which is associated with DNA cleavage during apoptosis. Induction was seen within 24 hours of drug treatment (Figure 4D–4E); suggesting γ-H2A.X may be a potential future biomarker of patient responsiveness to this drug.

### Tefinostat is synergistic with cytarabine

**Figure 5:**
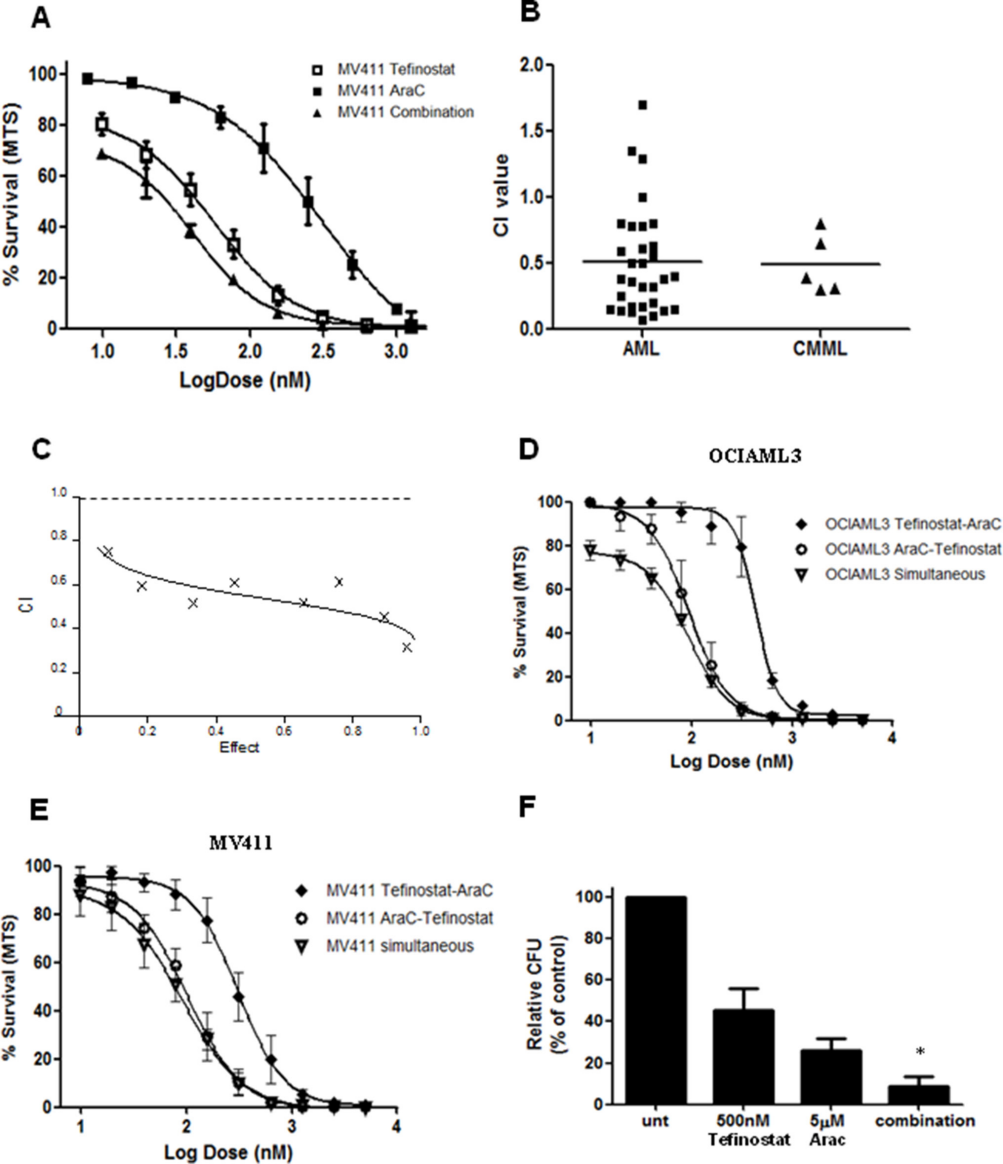
Tefinostat is synergistic with Cytarabine (**A**) Dose response curves for MV411 cells treated both singly and in combination for Tefinostat and AraC (dose ratio 1:10, Tefinostat:AraC). (**B**) Synergy analysis of tefinostat and AraC displayed as combination index (CI) values at EC50 dose affected at an optimal ratio of 1:10 (Tefinostat:AraC) in primary patient samples set up in triplicate (AML Mean combination index (CI) = 0.51, *n* = 31, CMML mean CI = 0.49, *n* = 5). (**C**) Representative CI response plot from primary AML showing CI values across a range of dose effects (fraction affected). Dose response curves for simultaneous and sequential dosing of drugs in (**D**) OCIAML3 and (**E**) MV411 cell lines. (**F**) Clonogenic assessment of synergy interaction in primary AML blasts (*n* = 4) following single agent and 1:10 ratio combination pulse treatment (*p < 0.04 MWU, between both single agents and combination colony growth).

To further inform clinical development of tefinostat we investigated the synergistic potential between tefinostat and the conventional cytotoxic agent cytosine arabinoside (AraC) at a fixed ratio of 1:10 (Tefinostat:AraC). *In vitro* synergy was demonstrated in combination experiments with clinically-relevant concentrations of tefinostat and cytarabine (AraC) firstly in MV411 cells (Figure 5A) and then in primary AML blasts (Figure 5B, Mean AML combination index (CI) = 0.51, *n* = 31 patient samples, Mean CMML CI = 0.53, *n* = 6 samples, CI at 50% fraction affected; values < 0.9 = synergistic, 0.9–1.2 additive, > 1.2antagonistic)). This synergism was observed across a range of drug effects (Figure 5C and Supplementary Table 3). Sequential administration of AraC followed by tefinostat did not increase synergistic effects beyond those observed with simultaneous application of the two agents (Figure 5D–5E); pre-treatment with tefinostat, however, was found to antagonise the combination response. Interestingly, tefinostat-responsive AMLs frequently stimulated NFκB p65 levels, commonly associated with drug resistance mechanisms, although these samples showed good synergy in combination with AraC (Supplementary Figure S4). Clonogenic assessment of single and combination pulse-treated AML blasts at an optimum ratio of 1:10 (Tefinostat: AraC) revealed a significant reduction in colonies in combination assays compared to with each agent used alone (*p* < 0.04).

## DISCUSSION

Although overall survival rates in AML have improved gradually over the last four decades this has been achieved largely through incremental improvements in supportive care and stem cell transplantation strategies with very little change in standard chemotherapy strategies over this time; minimal impact has been seen in clinical outcomes for older patients who represent the majority of newly-diagnosed cases. Similarly in CMML, a disease with an even higher median age of presentation, there is a considerable unmet need for non-toxic therapeutic strategies that are capable of altering the natural history of the disease. There is a strong pre-clinical rationale for HDAC inhibition in haematological malignancies, but systemic toxicities including gastrointestinal disturbances, myelosuppression, fatigue and insomnia have so far hindered the clinical update of HDAC inhibitors.

Here we report a series of *in vitro* experiments in which we confirm the monocytoid-lineage specificity of tefinostat in leukaemic cells obtained from patients with AML and CMML. We observed a dose-dependent induction of apoptotic response in myelo-monocytic cell types and a significantly greater drug sensitivity at sub-micromolar concentrations in this group compared to that seen in undifferentiated or non-monocytic patient samples. Esterase Sensitive Motif-mediated targeting of tefinostat and the resulting CHR-2847 accumulation in these cell types resulted in a 3.5–6.5 fold increase in anti-proliferative potency compared to the that seen using the untargeted t-butyl tefinostat analogue CHR-8185, in line with the previously-reported improved efficacy of tefinostat in comparison with other ‘non-targeted’ HDAC inhibitors such as vorinostat [23]. Lesser responses to tefinostat were seen in undifferentiated AML patient subgroups M0/M1; although sensitivity was observed in occasional undifferentiated samples with inherent HDAC inhibitor sensitivity this manifested as equal sensitivity to tefinostat and CHR-8185. Similarly, responses in M2 subtypes were variable and this correlated with the diversity of cellular sub-populations and CD14+ expression seen within this group.

Although mechanisms of action of HDAC inhibitors remain to be fully characterised, sensitivity to these agents has been linked to the apoptotic capability of each cell type. Differing combinations of apoptotic mediators, cell cycle abnormalities and differentiation blocks between patient samples make it difficult to predict cellular outcome to HDAC inhibitor treatment, although previous studies have shown that monocytic AML cell types such as the M5 cell line THP1 have greater apoptotic sensitivity to the HDAC inhibitor vorinostat than other non-monocytic AML cell lines [24]; this has been potentially linked to activity of Bcl-2 family members [25]. Previous studies of the HDAC inhibitor panobinostat have shown induction of growth arrest and increase in myeloid transcription factor expression associated with differentiation in AML cells [26]. We found, however, that tefinostat induces apoptosis at sub-EC_50_ level doses, indicating a different mechanism of action to that of panobinostat. Other potential mechanisms of action include acetylation and degradation of key chaperone molecules such as HSP90 [27] which supports a multitude of leukaemic oncoproteins. We find total HSP90 protein levels to be unaffected by tefinostat exposure in primary AML cells (data not shown), although this does not exclude the possibility of a functional change in HSP90 activity.

High levels of hCE-1 expression were found to drive a significant increase in tefinostat efficacy as measured by growth inhibition assays (*p* = 0.001), and also strongly correlated with expression of the mature monocytoid marker CD14^+^. CD14^+^ is widely used to define a mature monocytic cell type; we and others find, however, that CD14 may also be low or absent from leukaemic monocytes as part of the heterogeneity associated with this disease [28]. We observed that whilst, in the majority of patients, CD14 levels correlated with increased hCE-1 expression, this was not exclusive, particularly in M2 AMLs where, as previously reported, high hCE-1 levels were occasionally observed in the absence of significant CD14 expression [29].

Induction of intracellular H3/H4 histone acetylation was observed in all tefinostat-responsive AML and CMML patient samples in contrast to previous studies where other agents such as entinostat, vorinostat and panabinostat induced only modest levels of H3/H4 acetylation and monocytic populations were found to be resistant to HDAC inhibitory treatment [23], [30], [31]. hCE-linked targeting of tefinostat appears to circumvent this issue, with CD14+ monocytic AML sub-populations and CMML patient samples, as expected, showing the highest levels of drug response. Additionally, we observed preservation of normal bone marrow CD34^+^ cells and clonogenic function at AML-toxic doses of tefinostat in contrast to other HDAC inhibitors used in similar studies where significant increases in acetylation were observed in normal CD34+ cells [32, 33]. This finding emphasises the potential for limiting off-target systemic toxicities by harnessing hCE-1-dependent HDAC inhibitory activity in malignancies involving cells of monocytoid lineage (AML-M4, AML-M5 and CMML).

As yet a strong correlation between hyperacetylation and clinical response to HDAC inhibitors remains to be fully established. HDAC inhibitory efficacy is also reported to be dependent on accumulation of DNA damage [15], replication stalling [17], and apoptotic induction [34]. AML cases with leukaemia-associated fusion proteins are DNA damage repair deficient and therefore may be more resistant to HDAC inhibition [35]. We found no association, however, between tefinostat sensitivity and any patient characteristics such as cytogenetic group. We demonstrated that tefinostat induces the DNA damage marker γ-H2A.X in the majority of responsive patient samples similarly to that reported for other HDAC inhibitors such as vorinostat [36], suggesting that this may be a potential biomarker of patient response. Our data suggest that tefinostat induces both apoptosis and DNA damage accumulation within 24 hours of treatment in a majority of samples which may predict greater clinical response to this HDAC inhibitor. HDAC inhibition has also previously been demonstrated to signal pro-survival pathway feedback in the form of hyperacetylation of p65 NFκB signalling [37]; dual inhibition of HDAC and NFκB may potentiate efficacy in AML [38]. We observed a transient increase in NFκB p65 induction following tefinostat treatment, although this did not appear to impair γ-H2A.X or apoptotic induction in our sample cohort.

Another putative mechanism of action of HDAC inhibitors in single and combination studies is that of oxidative injury [16, 37, 39], This process is driven through NOX-mediated ROS induction which has been shown by our group to be constitutively active in myelomonocytic AML subtypes [40], and which may, in part, contribute to the greater efficacy of tefinostat in these patients. Although HDACis have been previously reported to selectively target FLT3 ITD-mutant proteins for degradation in AML [41] and to enhance FLT3 inhibition in cell lines [42, 43], we found no association between *in vitro* sensitivity to tefinostat and FLT3-ITD mutation status in our primary sample cohort (Supplementary Table S1).

As HDAC inhibition may result in a reduction of cells in S-phase, concerns have arisen that combination with traditional chemotherapeutic agents such as AraC may be antagonistic and that sequential administration may be required to optimise response [30, 37]. Our combination studies suggested that simultaneous dosing with tefinostat and AraC may be the most effective therapeutic regime and clonogenic synergy assays suggest this combination is also capable of targeting proliferative subsets within monocytoid AMLs. Pre-dosing with AraC did not significantly improve efficacy of the combination studies and pre-treatment with tefinostat produced an antagonistic response. Primary AML blasts demonstrated consistently high degrees of synergy in all primary samples tested (CI = 0.5, *n* = 36). This synergistic interaction with AraC (a principal component of AML cytotoxic regimens for many years) warrants further larger scale evaluation as a prelude to formal clinical evaluation.

The effective *in vitro* targeting of HDAC inhibition in monocytoid AML and CMML described above, combined with encouraging early signs of clinical activity (coupled to selective HDAC inhibition) in the absence of tefinostat-related toxicity in the Ossenkoppele study, provides a compelling case for further clinical evaluation of tefinostat in larger studies in monocytoid-lineage leukaemias. Rapid identification of suitable ‘monocytoid’ patients at diagnosis will present some challenges for trial design and will place an emphasis on close interactions between laboratory and clinic. Of the two CMML patients treated in the phase I study, one achieved a bone marrow complete response (CR) at relatively small doses and moving forward MONOCLE, a single-arm phase 2 study of tefinostat monotherapy in CMML (EudraCT 2015-002281-23) is shortly to commence recruitment at sites within the UK.

## METHODS

### Cell culture reagents and patient samples

AML samples were collected with informed consent from newly-diagnosed patients entering UK NCRI AML15, 16 and 17 studies (Ethics Reference Numbers 2005-001149/40, 2005-002847/14, 2007-003798/16) (Supplementary Tables S1 and S2) in accordance with the Declaration of Helsinki. CMML samples were obtained with informed consent under the MDSBio Sample Collection Study (Ethics Reference Number 06/Q1606/110). Mononuclear cells were purified by Ficoll density gradient followed by CD45 staining (AML samples with < 70% blast content following purification were excluded from further analysis). AML/CMML cells were cultured in IMDM (Sigma) with 10% FCS (Biosera). Normal bone marrow MNCs were purchased from Lonza and CD34^+^ cells were isolated using MiniMACS CD34 columns (Miltenyi Biotec) according to the manufacturer's instructions. HL60, MV411, OCIAML3 and THP-1 cell lines were cultured as recommended by ATCCLGC (http://www.lgcstandardsatcc.org). All cultures were incubated at 37°C, 5% CO_2_. Tefinostat (CHR-2845) and its N-butyl analogue control CHR8185 were supplied by Chroma Therapeutics (Abingdon, UK). Cytarabine was obtained from Sigma.

### Western blot analysis

Sample preparation of lysates and western blotting was performed as previously described [47]. Blots were probed with: γ-H2A.X Ser139 (Cell Signalling), hCE-1 (Lifespan biosciences). Equal loading was confirmed by Actin (Abcam). Quantification of proteins was carried out using AIDA image analyser v4.22 (Raytest, Straubenhardt, Germany).

### Drug response and cell viability assays

*In vitro* cell survival/drug response assays were set up with serial dilution of drugs in 96 well plates with 8 × 10^4^ cells per well. Cell viability was assessed by Cell Titre 96 Aqueous One MTS reagent (Promega) at 48 Hrs according to the manufacturer's instructions. Samples with less than 70% viability at 48 hours in untreated samples were excluded from analysis. For cytosine arabinoside (AraC) synergy experiments, dose response assays were set up in triplicate for single and fixed ratio combination treated AML cell lines and primary samples using clinically-relevant AraC concentrations. For sequential drug dosing cells were pre-treated for 24 Hrs with either Tefinostat or AraC prior to addition of the second drug. For sub-population analysis of drug response assays, cells were harvested after 48 h, surface stained with anti-CD45, CD14, CD64 and resuspended in 1 μg/ml 7aminoactinomycin D (7AAD) to determine viable cells remaining by flow cytometry using a FACSCalibur^®^ cytometer (BD Biosciences, Oxford, UK). Triplicates were averaged and dose response curves inputted to Calcusyn to generate EC_50_ and combination index values (Calcusyn v2.0 software, Biosoft, Cambridge, UK). Annexin V positivity was measured using the Annexin V Apoptosis Detection Kit (eBioscience, Hatford, UK) according to the manufacturer's instructions.

### Intracellular flow cytometry

Cells were prestained for 20 min with surface markers CD14, CD64, CD45 (BD Biosciences), then fixed in phosflow lyse buffer (BD biosciences) for 10 mins at 37°C before washing (PBS/0.5%BSA/0.1 NaAzide) and permeabilising in phosflow permeabilisation buffer II (BD biosciences) for 30 mins on ice. Primary antibodies were added for 45 mins at room temperature (RT) anti-hCE-1 (Lifespan biosciences LS-105283) or IgG control (Novous biological NB810-56910). For assessment of modulation of intracellular acetylation, cells were pre-incubated for 6 hours with Tefinostat prior to surface staining and fixation. Primary antibody Ac-k-103 (Cell signaling #9681) and IgG control (Mouse IgG2a ×0943 DAKO) were used at 200 ng/ml. Finally, cells were washed twice and incubated for 45 mins at RT with PE conjugated secondary antibodies goat-anti-rabbit or goat-anti-mouse (hCE-1 and Ak-k-103 respectively) prior to FACS analysis.

### Colony assays

CD34^+^ NBM cells and AML samples with > 70% CD14^+^ were plated in triplicate at 1 × 10^3^ (NBM) and 5 × 10^4^ (AML) cells per dish in semi-solid medium (Methocult Optimum H4034, Stem Cell Technologies, Location) in the presence of increasing doses of Tefinostat or vehicle control and cultured for 14 days at 37°C, 5% CO_2_ prior to colony counting. For synergistic assays, AML blasts were pulse treated with single agent (1 μM tefinostat, 10 μM AraC) or a 1:10 ratio (Tefinostat:AraC) prior to plating out in methocult.

### Statistical analysis

Multivariable analyses were performed using either logistic or Cox's proportional hazard regression methods adjusted for age, white blood cell count, cytogenetic group, performance status, de novo/secondary disease and sex. For all other results, differences between mean values were compared by Minitab v13 (Minitab Inc. PA, USA) using Mann WhitneyU or paired *t*-test.

## SUPPLEMENTARY MATERIALS FIGURES AND TABLES


