# Supplementary material for: The targeted histone deacetylase inhibitor tefinostat (CHR-2845) shows selective in vitro efficacy in monocytoid-lineage leukaemias
Source: Oncotarget. 2016 Feb 25;7(13):16650–62. doi: 10.18632/oncotarget.7692 (PMC4941341; doi:10.18632/oncotarget.7692)
Supplement: Supplementary file 1 [file oncotarget-07-16650-s001.pdf]

The targeted histone deacetylase inhibitor tefinostat (CHR-2845) shows selective *in vitro* efficacy in monocytoid-lineage leukaemias

Supplementary Materials

Supplementary Table S1: Relationship between patient characteristics and Tefinostat EC50

AML Patient Characteristic	Total	*P* value	AML Patient Characteristic	Total	*P* value
Overall	66		Performance Status		
			WHO 0	37	
**Trial**ǂ			WHO 1	19	
AML15	29		WHO 2	4	
AML16 (int)	4		WHO 3–4	6	0.7**
AML17	33	0.4*			
			FAB status		
**Age**			M0	4	
0–14	0		M1	6	
15–29	7		M2	8	
30–39	6		M4	23	
40–49	14		M5	17	
50–59	20		Unknown	8	0.009**
60–69	18				
70 +	1		Cytogenetics		
Median (range)	54.5 (16–75)	0.3**	Favourable	10	
			Intermediate	46	
**Sex**			Adverse	5	
Female	26		Unknown	5	0.9**
Male	40	0.16*			
			FLT3-ITD Mutation status		
**Diagnosis**			ITD WT	42	
de Novo	58		ITD Mutant	17	
Secondary	8	0.9*	Unknown	7	0.11*
**WBC**			NPM1 Mutation status		
< 10	2		NPM1 WT	28	
10–49.9	29		NPM1 Mutant	33	
50–99.9	20		Unknown	5	0.5*
100 +	15				
Median (range)	55.3 (7.5–350.3)	0.6**	FLT3-TKD Mutation status		
			TKD WT	62	
			TKD mutant	4	0.7*

*Wilcoxon Rank-Sum/Kruskal-Wallis test for difference between groups; **: Spearman correlation coefficient for continuous data/ordered groups; ǂ Trials AML15, 16 and 17 patients were treated intensively up to 2 rounds:

ADE (Daunorubicin, Cytarabine, Etoposide), DA/DAT (Daunorubicin, Cytarabine/Daunorubicin, Cytarabine, Thioguanine), FLAG-Ida (Fludarabine, Cytarabine, Idarubicin, G-CSF) (follow-up complete to 1/1/2015).

Supplementary Table S2: CMML patient characteristics

CMML Patient Characteristics	Total
Overall	7
**WHO Classification**	
CMML1	5
CMML2	2
**FAB Classification**	
Myelodysplastic (WBC < 13)	6
Myeloproliferative (WBC > 13)	1
**Age**	
0–59	0
60–69	2
70+	5
Median (range)	76 (68–84)
**Sex**	
Female	1
Male	6
**Diagnosis**	
de Novo	7
Secondary	0
**WBC**	
< 10	5
10–49.9	2
50–99.9	0
100+	0
Median (range)	4.29 (2.91–33.06)
**Cytogenetics***	
Favourable	5
Intermediate	0
Adverse	2

ǂTrial MDSBio1 patients enrolled to identify novel biomarkers of MDS.*Cytogenetic risk stratified by CMML specific risk classification, Such et al 2013

Supplementary Table S3: Synergistic analysis of tefinostat and araC interactions in cell line and primary material

Cell line	EC50 (nM) Simulataneous	EC50 (nM) Tefinostat pre-treatment	EC50 (nM) AraC pre-treatment
OCIAML3	88 +/− 3.2 (Ave CI = 0.67)	453 +/− 5.7	93 +/− 7.1
MV411	93 +/− 6.1 (Ave CI = 0.81)	332 +/− 7.7	107 +/− 10.5

AML Patient Sample	CI at ED50	CI at ED75	CI at ED90	CMML Patient Sample	CI at ED50	CI at ED75	CI at ED90
1	0.99	0.34	0.17	1	0.41	0.36	0.31
2	0.74	1.1	1	2	0.29	0.29	0.3
3	0.29	0.26	0.25	3	0.9	0.6	0.35
4	0.93	0.75	0.65	4	0.31	0.31	0.56
5	0.13	0.1	0.08	5	0.65	0.15	0.19
6	0.48	0.29	0.18				
7	0.68	0.54	0.43				
8	0.84	0.67	0.55				
9	0.5	0.19	0.07				
10	0.24	0.18	0.15				
11	0.4	0.48	0.59				
12	0.21	0.19	0.2				
13	0.71	0.61	0.52				
14	0.14	0.08	0.08				
15	0.43	0.47	0.53				
16	1.1	0.76	0.5				
17	0.2	0.62	1.1				
18	0.8	0.78	0.89				
19	0.45	0.43	0.43				
20	0.14	0.12	0.1				
21	0.37	0.29	0.26				
22	0.99	0.52	0.45				
23	0.12	0.2	0.34				
24	1.2	0.87	0.68				
25	0.86	0.98	1.1				
26	1.4	0.6	0.35				
27	0.95	0.74	0.59				
28	0.4	0.25	0.16				
29	0.8	0.63	0.54				
30	0.57	0.5	0.44				
31	1.65	1.1	1				

(CI values < 0.9 = Synergistic).

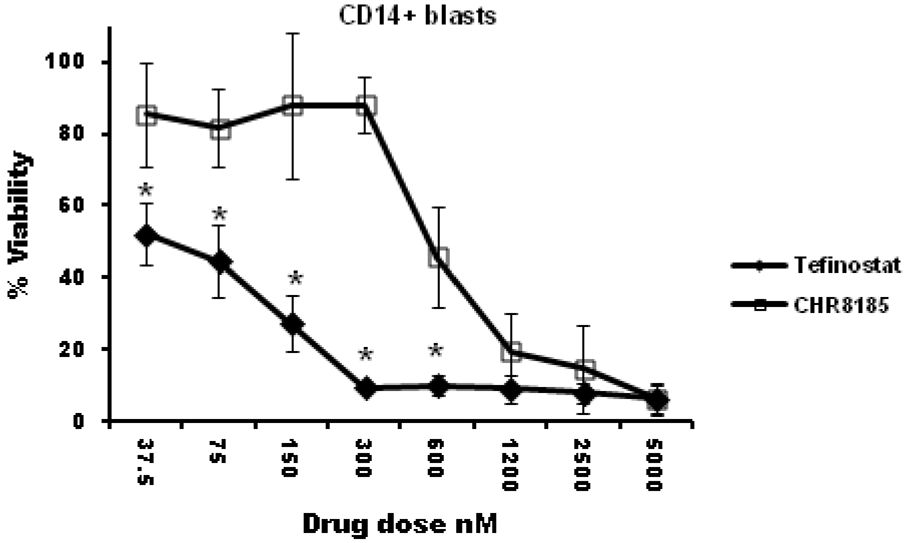

Supplementary Figure S1: Myelo-monocytic specificity of Tefinostat is demonstrated by 7AAD viability assays following 48 hrs dosing of Tefinostat compared to t-butyl analogue CHR8185 (n = 4, *p = 0.015, MWU).

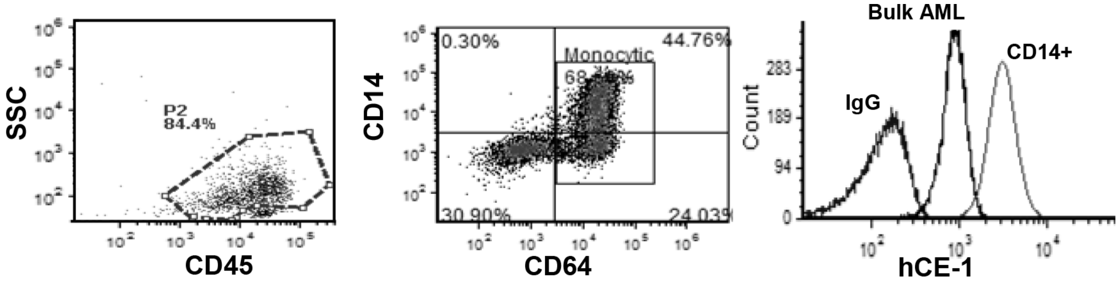

Supplementary Figure S2: Example flow data for Intracellular hCE-1 staining in a primary monocytic AML sample with cell surface markers CD45, CD14 and CD45 co-staining. Both bulk AML and CD14^{+} only AML populations were pre-gated by SSC/CD45 and sub-populations analysed for intracellular hCE-1 staining.

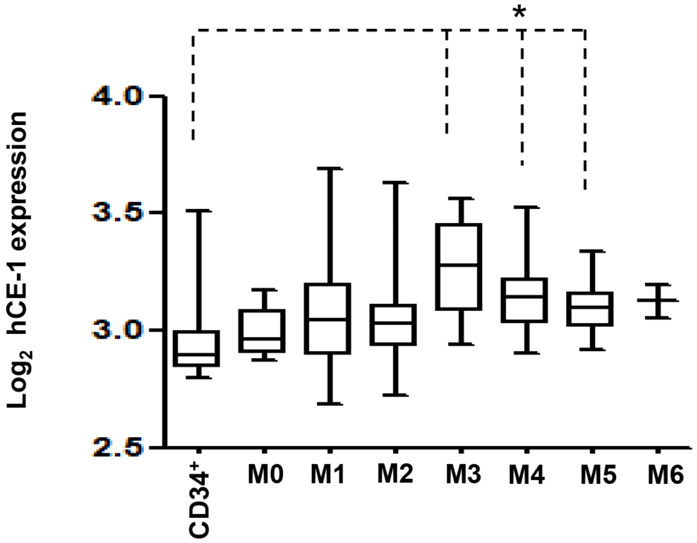

Supplementary Figure S3: Normalised expression of hCE-1 mRNA (*n* = 130 primary AMLs) across FAB groups compared to NBM CD34^{+} cells using the Affimetrix human U133A 2.0 array chip. *Kruskal Wallis+Dunn's $p < 0.01$. (GEO accession no. GSE13204. Haferlach et al J Clin Oncol. 2010 May 20; 28:2529–37).

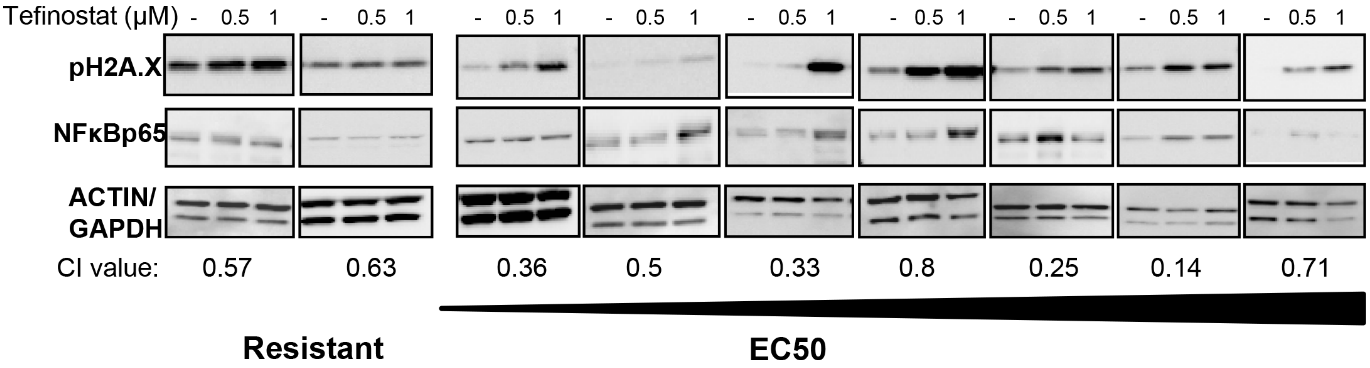

Supplementary Figure S4: Western blot analysis of NFκBp65 induction following 24 hrs drug treatment with tefinostat in 9 primary AML samples.
